# Establishment and Characterization of a Cell Line (S-RMS1) Derived from an Infantile Spindle Cell Rhabdomyosarcoma with *SRF-NCOA2* Fusion Transcript

**DOI:** 10.3390/ijms22115484

**Published:** 2021-05-22

**Authors:** Marta Colletti, Angela Galardi, Evelina Miele, Virginia Di Paolo, Ida Russo, Cristiano De Stefanis, Rita De Vito, Martina Rinelli, Andrea Ciolfi, Biagio De Angelis, Angelica Zin, Alessandro Guffanti, Maria Cristina Digilio, Antonio Novelli, Rita Alaggio, Giuseppe Maria Milano, Angela Di Giannatale

**Affiliations:** 1 Department of Pediatric Hematology/Oncology and Cell and Gene Therapy, Bambino Gesù Children’s Hospital, IRCCS, 00165 Rome, Italy; angela.galardi@opbg.net (A.G.); evelina.miele@opbg.net (E.M.); virginia.dipaolo@opbg.net (V.D.P.); ida.russo@opbg.net (I.R.); biagio.deangelis@opbg.net (B.D.A.); giuseppemaria.milano@opbg.net (G.M.M.); angela.digiannatale@opbg.net (A.D.G.); 2 Histology-Core Facility, Bambino Gesù Children’s Hospital, IRCCS, 00165 Rome, Italy; cristiano.destefanis@opbg.net; 3 Pathology Unit, Department of Laboratories, Bambino Gesù Children’s Hospital, IRCCS, 00165 Rome, Italy; rita.devito@opbg.net (R.D.V.); rita.alaggio@opbg.net (R.A.); 4 Laboratory of Medical Genetics, Translational Cytogenomics Research Unit, Bambino Gesù Children Hospital, IRCCS, 00165 Rome, Italy; martina.rinelli@opbg.net (M.R.); mcristina.digilio@opbg.net (M.C.D.); antonio.novelli@opbg.net (A.N.); 5 Genetics and Rare Diseases Research Division, Bambino Gesù Children’s Hospital, IRCCS, 00165 Rome, Italy; andrea.ciolfi@opbg.net; 6 Institute of Pediatric Research Città Della Speranza and Laboratory of Solid Tumors, Clinic of Pediatric Hematology-Oncology, University of Padova, 35129 Padova, Italy; angelica.zin@unipd.it; 7 Nico Innovagroup S.r.l., 25125 Brescia, Italy; alessandro.guffanti@nicoinnovagroup.com

**Keywords:** spindle cell, rhabdomyosarcoma, *SRF-NCOA2*, cell line

## Abstract

*Background*: Spindle cell rhabdomyosarcoma (S-RMS) is a rare tumor that was previously considered as an uncommon variant of embryonal RMS (ERMS) and recently reclassified as a distinct RMS subtype with NCOA2, NCOA1, and VGLL2 fusion genes. In this study, we established a cell line (S-RMS1) derived from a four-month-old boy with infantile spindle cell RMS harboring *SRF-NCOA2* gene fusion. *Methods*: Morphological and molecular characteristics of S-RMS1 were analyzed and compared with two RMS cell lines, RH30 and RD18. Whole genome sequencing of S-RMS1 and clinical exome sequencing of genomic DNA were performed. *Results*: S-RMS1 showed cells small in size, with a fibroblast-like morphology and positivity for MyoD-1, myogenin, desmin, and smooth muscle actin. The population doubling time was 3.7 days. Whole genome sequencing demonstrated that S-RMS1 retained the same genetic profile of the tumor at diagnosis. A Western blot analysis showed downregulation of AKT-p and YAP-p while RT-qPCR showed upregulation of endoglin and GATA6 as well as downregulation of TGFßR1 and Mef2C transcripts. *Conclusion*: This is the first report of the establishment of a cell line from an infantile spindle cell RMS with SRF-NCOA2 gene fusion. S-RMS1 should represent a useful tool for the molecular characterization of this rare and almost unknown tumor.

## 1. Introduction

Rhabdomyosarcoma (RMS) is the most common type of soft tissue sarcomas in children, accounting for 5%–8% of all pediatric tumors, and it may be congenital in 0.4%–2% of the cases [1]. RMS has been traditionally classified into two major subgroups based on histopathologic and molecular criteria: embryonal RMS (ERMS) and alveolar RMS (ARMS). ERMS is more frequent and occurs in younger patients with a more favorable prognosis, whereas ARMS accounts for around 20% of all cases, and it is often diagnosed in older children with a highly aggressive clinical course. About 80% of the ARMS is associated with a characteristic translocation of the FOXO1 gene at 13q14 with PAX3 at 2q35 or less commonly PAX7 at 1p36 [2]. Spindle cell RMS was originally described as a pediatric variant of ERMS characterized by a uniform proliferation of elongated spindle cells mimicking smooth muscle fibers, generally arising in the paratesticular, head, and neck regions and is associated with a more favorable prognosis [3,4]. However, in adults, SRMS appeared to have a more aggressive clinical course [5,6]. After the identification of the sclerosing subtype of RMS [5], it became evident that this entity, characterized by small cells with minimal rhabdomyoblastic differentiation within a sclerotic stroma that form cords, nests, and microalveoli, represented a morphologic continuum with spindle rhabdomyosarcoma [5,6,7]. Since the 2013 WHO classification, these tumors are considered as a single rhabdomyosarcoma type [8], and they encompass spindle cell/sclerosing rhabdomyosarcomas with MyoD1 mutations, occurring in older children and young adults with a highly aggressive clinical behavior and infantile spindle cell rhabdomyosarcomas. This subgroup was originally described in 1994 under the name of rhabdomyofibrosarcoma [9]. Due to its rarity, this entity had been ignored until 2013, when Mosquera and colleagues identified recurrent NCOA2 (nuclear receptor coactivator) gene rearrangements in a small subset of congenital/infantile spindle cell RMS involving *SRF* and *TEAD1* genes [10]. In 2016, Alaggio and collaborators reported additional gene fusions in infantile SRMS including *TEAD1-NCOA2*, *VGLL2-CITED2*, and *VGLL2-NCOA2* fusions [11].

To date, only two SSRMS cell lines derived from residual tumor after neoadjuvant chemotherapy have been established and characterized. One is NCC-ssRMS-C1, which derived from a 17-year-old female [12], while the other, SRH, is from a 24-year-old female [13].

The establishment and characterization of primary cancer cell cultures from fresh surgically resected tissue samples are important preclinical tools for investigating tumor behavior, especially in extremely rare forms of cancers [14,15]. The possibility of having an ex vivo culture of patient-derived tumor samples allows us to obtain an in vitro system where the different cellular phenotypes and the heterogenicity of the subpopulations present in the mass are preserved. Moreover, this preclinical tool is useful for making comparative evaluations with respect to the primitive mass to evaluate the response of human cancer cells to drugs or to improve personalized treatments [16].

Herein, we report the establishment and characterization of a new cell line named S-RMS1 that was derived from a four-month-old male affected by a congenital S-RMS harboring the *SRF-NCOA2* gene fusion. To our knowledge, this is the first cell line established from an infantile S-RMS.

## 2. Results

### 2.1. Establishment of S-RMS1 Cell Line

**Figure 1:**
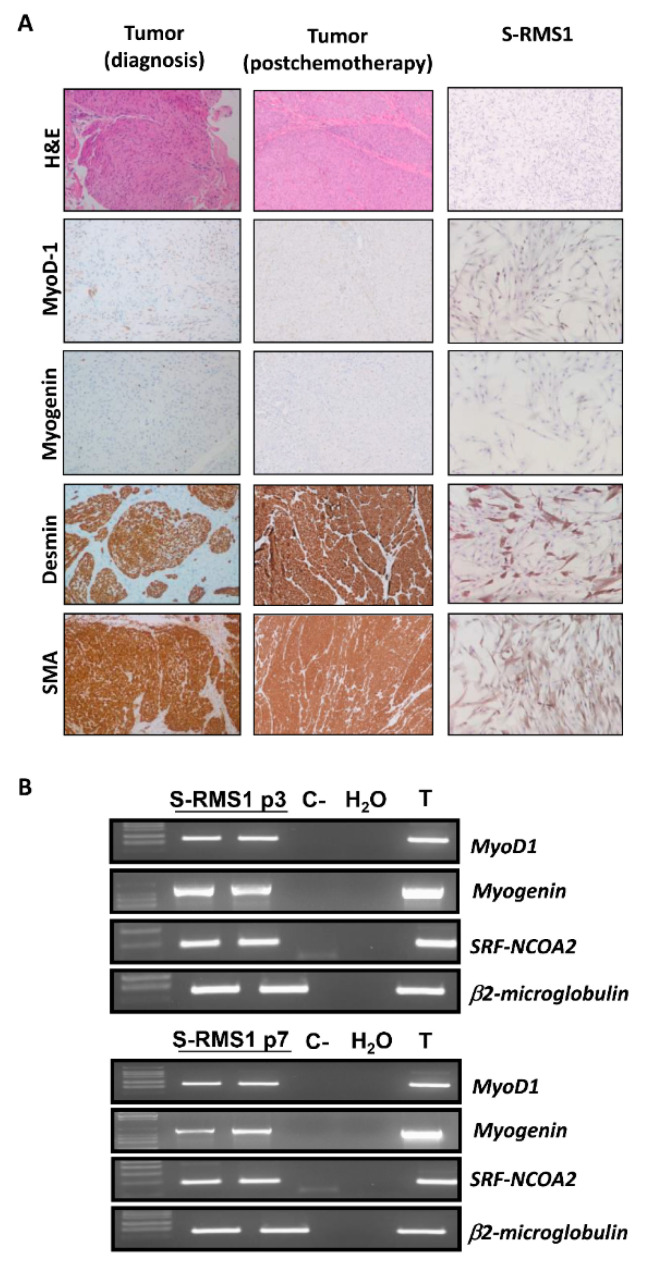
Establishment of S-RMS1 cell line. (**A**) IHC characterization of tumor at diagnosis, postchemotherapy, and S-RMS1 cell line. S-RMS1 cell line showed positivity for MyoD-1, myogenin, desmin and smooth muscle actin (alpha-SMA) (magnification 20×), such as primary tumor pre- and postchemotherapy. Comparison of S-RMS1 morphology with RD18 and RH30. (**B**) Identification of fusion transcript *SRF-NCOA2* in S-RMS1 at two different passages (p3 and p7). Reverse transcriptase polymerase chain reaction detection of MyoD1 (264 bp), myogenin, and SRF-NCOA2 chimeric transcript (93 bp). β2-microglobulin was used for normalization. cDNA obtained from patient primary tumor (T) was used as positive control whereas cDNA from T lymphoblastoid CEM cell line (C-) was taken as negative control.

The S-RMS1 cell line was established from the resected fresh tumor obtained through surgery, after neoadjuvant chemotherapy, from a four-month-old boy diagnosed at birth with an infantile S-RMS. The needle biopsy of the lesion at the diagnosis showed at histology a densely cellular tumor that was composed of elongated cells, with eosinophilic cytoplasm and ovular nuclei, with mild pleomorphism and finely dispersed chromatin. The cells were arranged in intersecting fascicles with a vaguely whorled pattern. Rhabdomyoblastic differentiation was almost undetectable. The mitotic rate was low, and necrosis was absent. Immunohistochemically, the tumor cells exhibited a diffuse positive nuclear staining for MyoD-1, cytoplasmic staining for desmin, and smooth muscle actin (SMA). Myogenin was expressed only focally, while the S-100 protein was negative (Figure 1A left). The median percentage of Ki67 was 15%. Finally, on RNA isolated from the primary lesion, we identified the SRF-NCOA2 rearrangement (chr6:43146615, chr8:71068210) and performed a next generation sequencing based on the Archer FusionPlex Sarcoma kit (ArcherDX, Boulder, CO, USA), using a method recently described [17]. Sanger sequencing (data not shown) was also performed on the primary lesion, and we found a complete overlap between our sequence and the rearrangement sequence shown in the work of Mosquera and collaborators [10], which described recurrent *NCOA2* gene rearrangements in a congenital/infantile case of spindle cell RMS involving the *SRF* gene. NCOA2 belongs to the family of p160 steroid receptor coactivators, which are not transcription factors but interact with ligand-bound nuclear receptors to recruit histone acetyltransferases and methyltransferases facilitating chromatin remodeling [18]. SRF is a transcription factor highly expressed in the skeletal muscle, where it controls the transcription of genes involved in muscle differentiation and sarcomeric protein encoding [19] (Supplementary Materials, Figure S1).

In consideration of all findings, a diagnosis of infantile S-RMS was given. The histology of the surgical excision of the mass, which was performed postchemotherapy, confirmed an infantile spindle cell RMS, with a necrosis less than 1%. The immunophenotype was preserved (Figure 1A center). The S-RMS1 cell line was positive for MyoD-1, desmin, SMA and was weak for myogenin (Figure 1A right). RT-PCR confirmed SRF-NCOA2 fusion both in the tumor evaluated at diagnosis (T) and in the S-RMS1 cell line (Figure 1B) at different passages (passage 3 above panel, passage 7 below panel) as well as positivity for MyoD-1 and myogenin.

### 2.2. Whole Genome Resequencing and Bioinformatic Analysis of the S-RMS1 Cell Line

**Figure 2:**
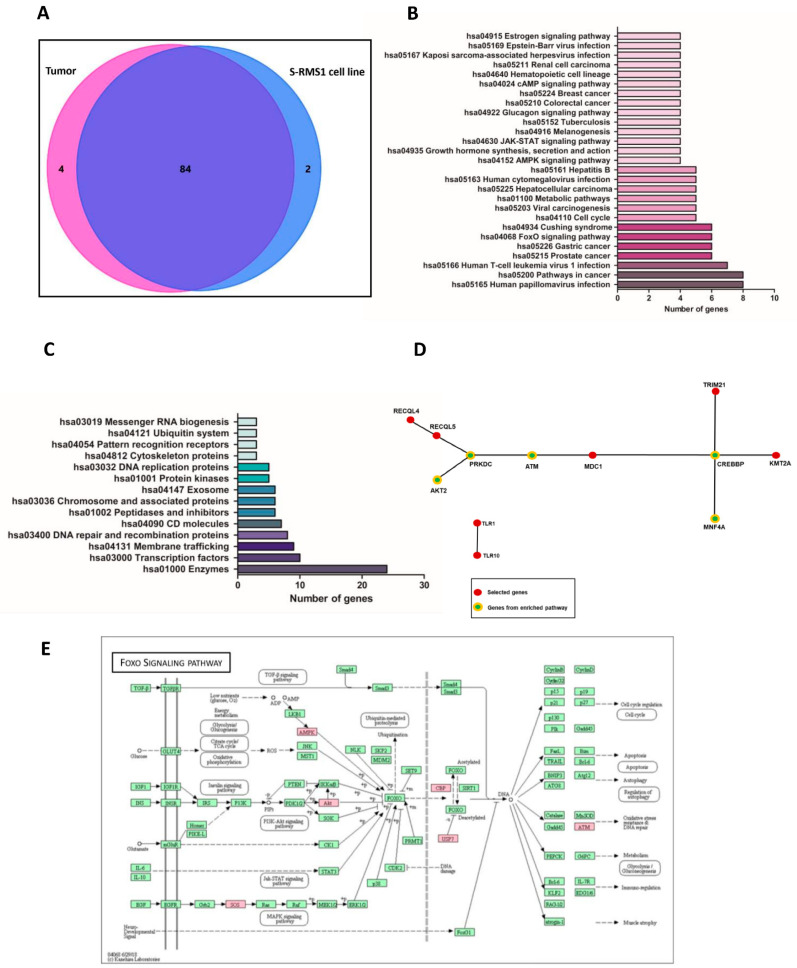
Tumor and S-RMS1 cell line whole genome resequencing and bioinformatic analysis. (**A**) Venn diagram analysis performed with FunRich software. The pink circle represents the tumor at diagnosis dataset; the blue circle represents the S-RMS1 dataset. The intersection of the two circles represents overlapping genes with variants (SNPs and indels) among the two datasets. (**B**) Gene ontology (GO) terms and KEGG pathways analysis using 84 common genes reporting genomic variants between the two datasets (tumor at diagnosis and S-RMS1 cell line). The graph shows pathways where at least 4 genes of the 84 that are common to the two samples were implicated. (**C**) Graphical representation of functional categories for the 84 common genes. Categories with at least 3 genes of the dataset are reported. (**D**) Interaction pathway performed with FunRich Software determined by the analysis of the 84 genes with genomic variants shared by the tumor at diagnosis and S-RMS1 cell line. Red circles represent the gene with at least one interaction and green circles the genes with more than one interaction belonging to enriched pathways. (**E**) FoxO signaling pathway in KEGG pathway enrichment. Red boxes indicate the genes presenting genomic variants from the list of 84 common genes.

Whole genome resequencing of DNA from the S-RMS1 cell line was compared to the tumor tissue at diagnosis, in order to investigate and quantify the overlap of the mutational status and the similarities of the two different samples. Extensive gene variant overlap was found between the tumor at diagnosis and at the derived S-RMS1 cell line, showing that this cell line is an exact representation of the patient tumor. The analysis generated a total of 903,430,644 and 1,264,129,174 sequence reads with 84.5% and 96.3% mappings to the genome of the tumor and of the S-RMS1 cell line, respectively. For S-RMS1, the sequencing identified 4,180,071 variants in a first instance: 3,571,256 single-nucleotide polymorphisms (SNPs) and 608,815 INDELs (312,524 small insertions, 296,291 small deletions). Among these, 11,641 were synonymous variants, 10,559 were nonsynonymous variants, 297 were splicing variants, 79 were stop-gain, 40 were stop-loss, and 389 were frameshift. Regarding the tumor, we found in a first instance 3,619,734 variants: 3,619,061 single-nucleotide polymorphisms (SNPs) and 673,734 INDELs (352,903 small insertions, 320,831 small deletions). Among these, 11,699 were synonymous variants, 10,675 were nonsynonymous variants, 309 were splicing variants, 81 were stop-gain, 39 were stop-loss, and 423 were frameshift. In both samples, the variants were distributed along the whole genome with the greater number of variants present on chromosome 2 and the lower number on chromosome Y (Supplementary Materials, Figure S2). From the analysis performed with the Genoox software, prioritizing data with the term “rhabdomyosarcoma” coupled with all the other genetics prioritization approaches evaluation of the damaging effect of the variant—stop-gain, start-gain, nonframeshift, start-loss, frameshift other, stop-loss, missense—reported the frequency of the identified SNP in public variant databases and so on. In particular, in the tumor at diagnosis, 88 genes were identified, whereas in the S-RMS1 cell line, 86 genes bearing variants were identified and, of these, 84 were common between the two samples, sharing also the same zygosity (Figure 2A, Supplementary Materials, Table S1). The comparison showed 4 and 2 unique variants in the tumor at diagnosis and in the S-RMS1 cell line samples, respectively (Table 1 and Table 2). Using gene ontology (GO) terms and a KEGG pathway analysis, we identified pathways where at least 4 of the 84 genes common to the two samples were implicated (Figure 2B), and interestingly between the pathways most represented were the hsa05200 pathways in cancer (8 genes: CREB binding protein, *CREBBP*; endothelial PAS domain protein 1, *EPAS*; AKT serine/threonine kinase 2, *AKT2*; mutS homolog 3, *MSH3*; RB transcriptional corepressor 1, *RB1*; SOS Ras/Rho guanine nucleotide exchange factor 2, *SOS2*; transcription factor 7 like 2, *TCF7L2*; telomerase reverse transcriptase, *TERT*), the hsa04068 FoxO signaling pathway (6 genes: *CREBBP*; *AKT2*; ATM serine/threonine kinase, *ATM*; protein kinase AMP-activated noncatalytic subunit gamma 3, *PRKAG3*; *SOS2*; ubiquitin specific peptidase 7, *USP7*), and the hsa04110 cell cycle (5 genes, cyclin dependent kinase inhibitor 1C, *CDKN1C*; *CREBBP*; *ATM*; protein kinase, DNA-activated, catalytic subunit, *PRKDC*; *RB1*). Of the 84 genes, 24 were coded for enzymes, 10 for transcription factors, 9 for proteins implicated in membrane trafficking, 8 for DNA repair and recombination proteins, 7 were CD molecules, 6 were peptidases and inhibitors, chromosomes, and associated proteins or exosomal proteins, 5 were protein kinases or DNA replication proteins, and 3 belonged to the functional categories of cytoskeleton proteins, pattern recognition receptors, the ubiquitin system, or messenger RNA biogenesis (Figure 2C). Interaction analysis performed by FunRich software identified a network described by 10 genes (*RECQL4*, *RECQL5*, *AKT2*, *PRKDC*, *ATM*, *MDC1*, *CREEBP*, *HNF4A*, *TRIM21*, *KMT2A*) and one composed by 2 genes (*TLR1* and *TLR10*) as seen in Figure 2D. Among these genes, a central role in this interaction network is occupied by *CREBBP* and followed by *ATM*, *PRKDC*, *AKT2*, and *HNF4A*. In Figure 2E, we show the KEGG pathway hsa04068 FoxO signaling pathway with the genes reporting SNP and INDELs in our analysis in red.

### 2.3. Clinical Exome Sequencing of Genomic DNA and Germline Variant Identification

Exome sequencing filtering and prioritization identified five potential candidate variants in different genes that were absent or present with an MAF < 0.01 in population databases (Table 3). The comparison between the S-RMS1 cell line and the genomic DNA of the patient revealed an overlap of five variants as associated with the patient’s clinical features. All variants were identified in the heterozygous condition in the patient and were found to segregate in one of the healthy parents.

The nonsense variant c.5221C>T, p.Gln1741* (rs781481160), which was localized in exon 39 of the *POLE* (NM_006231) gene had an allele frequency in all the samples of our account (AF%) of 0.0030. The substitution c.5221C>T, which is maternally inherited, causes the p.Gln1741* stop variant, which was predicted to result in a truncated protein. It was reported in the ClinVar database (RCV000602212.2) and classified as an uncertain significance variant (VUS) according to the ACMG guidelines. The maternal c.8428A>C variant in the *ATM* (NM_000051.3) gene caused the missense variant p.Lys2810Gln (rs730881325) in exon 58. The variant, with an AF% of 0.012, was reported in ClinVar (RCV000168380.10) and in the literature as a VUS variant, associated to a hereditary cancer-predisposing syndrome [20]. The maternal missense variant in the *TERT* gene (NM_198253.2) was localized in exon 2 and caused the protein substitution p.Pro308Thr. It has never been described in literature, but it is classified as a VUS by ACMG guidelines with an AF% of 0.0030. The only paternal variant in the first exon of *CDKN1C* (NM_000076.2), gene c.392_394delAGG, causes the in-frame indel variant p.Glu131del. The variant, with an AF% of 0 in our account according to the allele frequencies in reference population (MAF%), is not reported in literature, and it is also considered as a VUS variant. Finally, the *CREBBP* (NM_004380.2) variant in exon 31 caused the maternal variant c.5800T>C and the aminoacidic substitution p.Ser1934Pro (rs587783504). The missense variant was reported in ClinVar (RCV000145768.1) as a VUS variant associated to the Rubinstein–Taybi syndrome type 1 (OMIM 180849), a rare autosomal dominant genetic condition. After careful evaluation of the data detected by the sequencing carried out by an expert geneticist, none of the mutations were found to be certainly causative of the child’s pathology. In fact, all variants segregate from a healthy parent, determining incomplete expressiveness and penetrance, and possibly determining a multifactorial mechanism. The genes most likely to be implicated in this mechanism in our case are the *POLE*, *CDKN1C*, and *ATM* genes, while the etiological involvement of the *CREBBP* gene can be excluded as no clinical elements emerged for the Rubinstein–Taybi syndrome.

### 2.4. DNA Methylation Profiling of S-RMS1 Cell Line

To further characterize the S-RMS1 cell line, we performed DNA methylation profiling and compared it with both the tumor tissue (T) and 5 other samples of ERMS diagnosed at the Bambino Gesù Children’s Hospital and used as the control group (Table 4).

**Figure 3:**
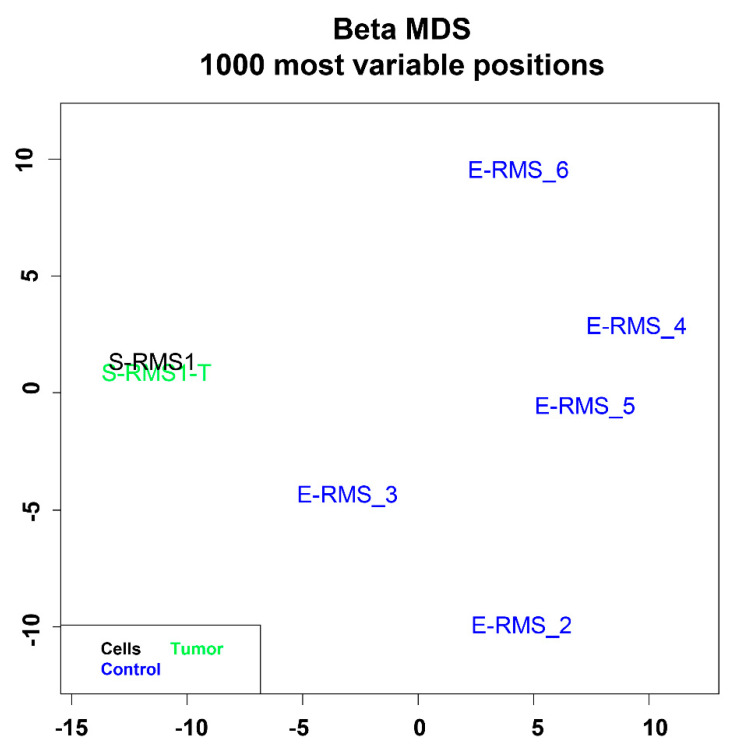
Genome-wide DNA methylation profiling on RMS samples and cell line. MDS (multidimensional scaling) analysis performed on the 1000 most variable probes of the whole genome DNA methylation data shows a close similarity between S-RMS1 cell line and tumor tissue (T). Color legend of the MDS plot as follows: S-RMS1 cell line (S-RMS1, black); S-RMS1 tumor (S-RMS1-T, green); E-RMS_2, 3, 4, 5, 6 embryonal rhabdomyosarcomas as controls (blue).

A multidimensional scaling (MSD) analysis performed on the 1000 most variable probes of the whole genome DNA methylation data shows a close similarity between S-RMS1 cell line at passage 6 and the tumor tissue (T), while there is no similarity with ERMS samples (Figure 3). This result suggests that S-RMS1 in vitro model retains the epigenetic signature of the original tumor, which is therefore to be considered relevant for basic and translational biology. The copy number variation (CNV) plot shows the rearrangement of chromosomes 6 and 8 in the pre- and postbiotic samples and in S-RMS1 cells to a lesser extent with a low mutational tumor burden (Supplementary Materials, Figure S3). The methylation data of the tumor samples and derived cell lines were firstly categorized using the recently introduced sarcoma classifier v12.2 (https://www.molecularneuropathology.org/mnp/classifier/9 (accessed on 23 April 2021) [21], which also generated copy number variation (CNV) plots. None of the tumor samples nor the primary cell lines were clustered in a defined methylation class. Looking at the raw scores, the first and second methylation classes were angioleiomyoma/myopericytoma (ALMO/MPC) and alveolar soft part sarcoma (ASPS), respectively, for the tumor specimens and ASPS (first) and rhabdomyosarcoma, embryonal (RMS-EMB) (second) for the cell line (Supplementary Materials, Figure S4).

Altogether, these data suggest that the DNA methylation profiling did not confirm nor orient the diagnosis for this case, probably due to the inexistence of a well-defined entity in the current classifier as evidenced by the very low raw and calibrated scores.

### 2.5. Morphology, Growth, and Molecular Characterization of S-RMS1 Cell Line

**Figure 4:**
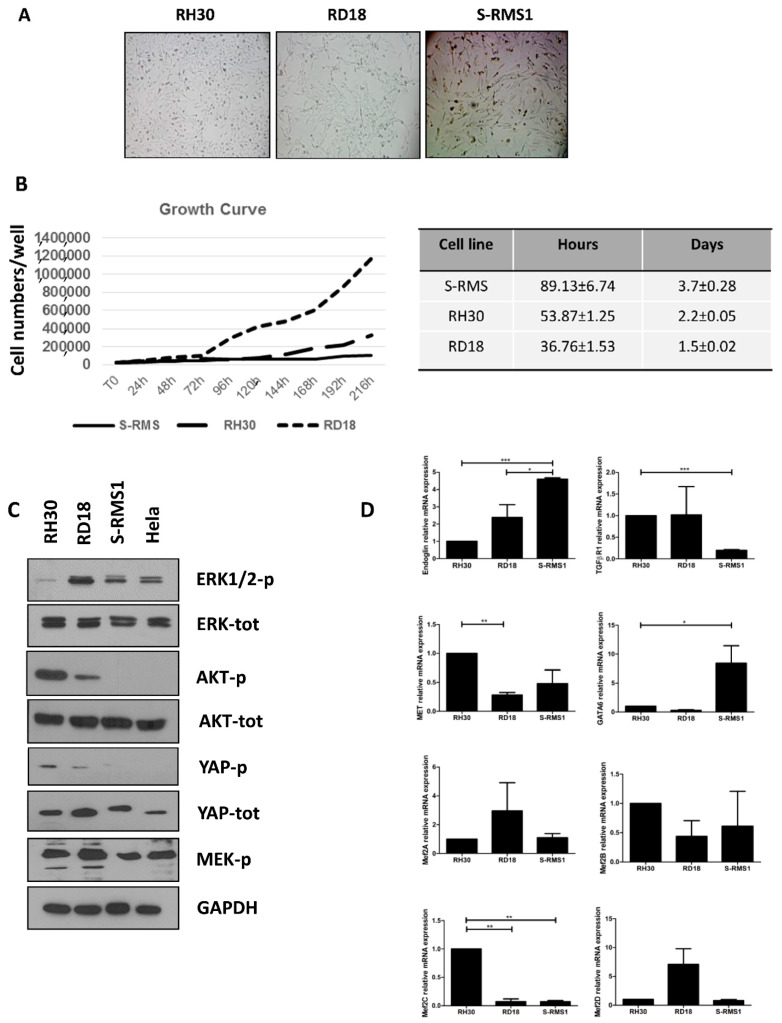
Characterization of S-RMS1 cell line. (**A**) Morphology of S-RMS1 compared with RH30 and RD18 cell lines (magnification 10×). (**B**) The population doubling time was calculated as reported and found to be of about 3.5 days for S-RMS1. (**C**) Western blot analysis of several pathways involved in rhabdomyosarcoma pathogenesis. (**D**) Real time qPCR of genes implicated in skeletal muscle differentiation (Mef2A, Mef2B, Mef2C, and Mef2D) and tumorigenesis (Endoglin, TGFß-RI, MET, and GATA6).

S-RMS1 cells were small in size with a fibroblast-like morphology, which is maintained during several passages of culture. The S-RMS1 cells were passaged near 10 times. In comparison with other two RMS cell lines, RH30 (alveolar) and RD18 (embryonal), their morphology is more elongated and fusiform (Figure 4A). In order to examine S-RMS1 proliferative capacity, RH30 and RD18 cells were plated in parallel while the same culture conditions were utilized. The number of cells was evaluated each day for 9 days. The growth curve examined at passage number 7 of the S-RMS1 cells displayed a doubling time of about 3.7 ± 0.28 days compared to RH30 and RD18, which had a doubling time of 2.2 ± 0.05 and 1.5 ± 0.02 days, respectively (Figure 4B).

The S-RMS1 cell line was then characterized for the expression of key signaling molecules involved in the pathogenesis of RMS, in comparison to the other RMS cell lines, RH30 and RD18. Interestingly, a Western blot analysis revealed that S-RMS1 presented a lower level of YAP-p and MEK-p in comparison with RH30 and RD18 (Figure 4C). Compared to RD18 and RH30, the gene expression analysis of the transcripts involved in skeletal muscle differentiation (Mef2A, Mef2B, Mef2C, Mef2D) and tumorigenesis (endoglin, TGFβRI, MET and GATA6) demonstrated a significantly higher level of endoglin (*p* < 0.0001), a marker of neovascularization in RMS [22] and GATA-6 (*p* < 0.05) in S-RMS1, while TGFβ1R was downregulated (*p* < 0.0001), and Mef2C, MET, Mef2A, Mef2B, and Mef2D were similarly expressed (Figure 4D).

### 2.6. Tumorigenic Properties of S-RMS1 Cell Line

**Figure 5:**
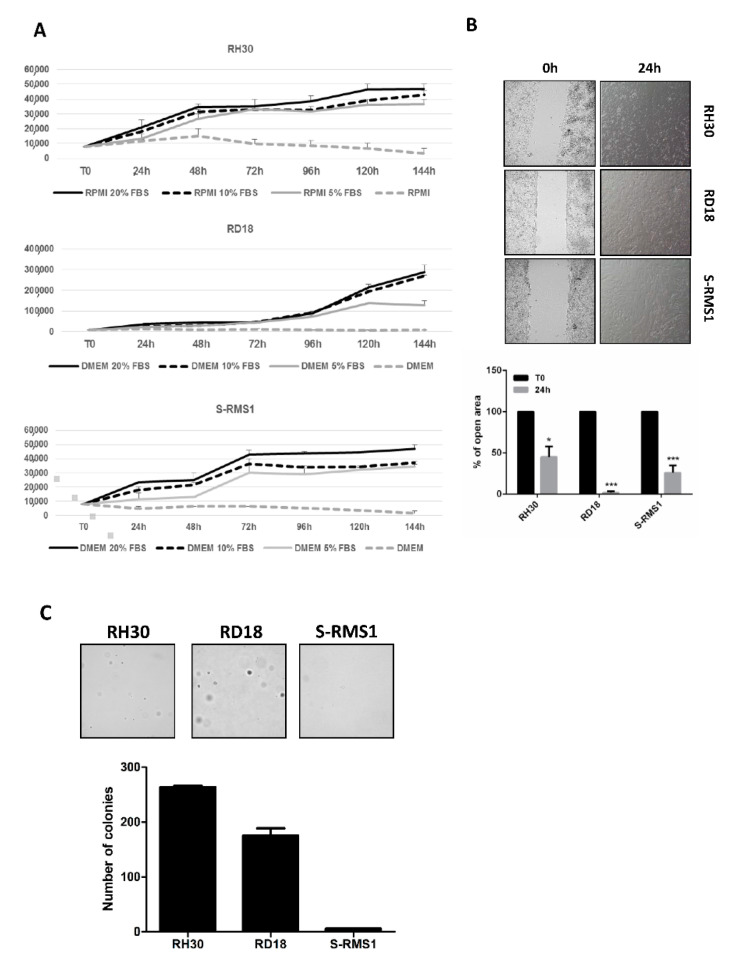
Tumorigenic properties of S-RMS1 cell line. (**A**) Serum independent growth of S-RMS1, RH30, and RD18 cells. (**B**) Representative phase contrast microscopy images of the migration assay at 0 and 24 h after gap creation. The histogram depicts the measurement in percentage of the total area between the wound edges of the scratch from at least five random fields per scratch from three independent experiments. Data are shown as mean ± SEM. * *p* < 0.05; *** *p* < 0.0001 Student’s *t*-test. (**C**) Clonogenic ability in anchorage-independent manner of S-RMS1, RH30, and RD18 cell lines. Histogram depicts the number of colonies per plate after 4 weeks of incubation, calculated as means ± SEM from three independent experiments.

In order to examine the autocrine growth of the S-RMS1 cell line, we assessed the S-RMS1 cell proliferation ability in serum-independent conditions in comparison to RH30 and RD18 cell lines (Figure 5A). In serum free media, about half of the S-RMS1 cells die in 24 h compared to RH30 and RD18, which remain half alive after 72 h and 48 h, respectively. To further characterize the tumorigenic properties of the S-RMS1 cell line, we evaluated the migratory potential of these cells in comparison with RH30 and RD18 in a wound-healing assay (Figure 5B). At 24 h after cell seeding, we observed 45.16% ± 12.8% for RH30, 1.9% ± 1.4% for RD18, and 26.01% ± 8.6% for S-RMS1 of open area, suggesting for S-RMS1 an intermediate migration rate with respect to RH30 and RD18 cells. To test the clonogenic ability of our cell line, we performed a colony formation assay in soft agar in an anchorage-independent manner. As shown in Figure 4C, after 4 weeks of growth in soft agar, S-RMS1 cells have the ability to form 5 ± 0.6 colonies, unlike the RH30 and RD18, in which we counted 263 ± 2.6 and 175 ± 12.9 colonies, respectively. This result is in line with the observation that S-RMS is associated with a more favorable behavior in children than ARMS or ERMS [11].

## 3. Discussion

First described in 1992 by Cavazzana et al. [3] as a variant of ERMS and occurring primarily in the paratesticular or head and neck region, spindle RMS has recently emerged as a standalone pathologic entity [23]. Three different groups are included: (i) MyoD1 mutated spindle cell/sclerosing RMS that mostly occurs in older children and adults, with highly aggressive behavior; (ii) infantile S-RMS, with NCOA2, NCOA1, and VGLL2 fusions and favorable prognosis; and (iii) a subset of tumors probably representing a spindle cell variant of ERMS. Thus, the term refers to morphological features but includes a heterogeneous group of molecularly different tumors. Since the identification of infantile S-RMS, RMS in the first few months of life is becoming an emerging field of interest in pediatric oncology because of the peculiarity of the molecular and pathologic features as well as the clinical implications related to the need of a minimally aggressive treatment. A recent study published by the French MMT committee reported 8 infants, aged less than 6 months, with a diagnosis of SRMS, and among these, three showed VGLL2-rearrangements, i.e., one NTRK-fusion and two (B)RAF-fusions [24]. This cohort, together with the one previously described by Alaggio et al. [11], confirmed the excellent outcome of these infantile spindle cell sarcomas with fibromatous/fibrosarcoma-like morphology and positivity for myogenin. More recently, well-differentiated RMS with variable spindle cell morphology and SRF fusions was reported, further expanding the morphologic and molecular spectrum of infantile RMS [25]. In the study, 12 out 13 patients were children and infants; the tumors were frequently located in the head and showed a benign behavior. These tumors were associated to the SRF-RELA fusion gene in addition to other genetic alterations, all involving SRF fused to CITED1, CITED2, NFKBIE, or NCOA2. Since there is no consensus for an optimal treatment for these RMS tumors, an excellent prognosis may support more conservative future strategies in this subset of patients. Our patient is still in complete remission after more than 3 years from the diagnosis despite a local relapse after 10 months from the stop therapy.

Due to the extreme rarity of this disease, only two cell lines that were derived SSRMS have been described until now. The first was derived from a 17-year-old female [12] and the second from a 24-year-old female [13], both affected by SSRMS and with MyoD1 mutation, which is a hallmark of tumor aggressiveness. Here, we report the isolation and characterization of the first human cell line derived from an infantile S-RMS harboring the *SRF-NCOA2* gene fusion. This is the second case of infantile spindle cell RMS with SRF-NCOA2 gene fusion along with the one described by Mosquera et al. [10].

The established cell line, named S-RMS1, showed a spindle-shaped morphology, which was maintained during several passages in culture. S-RMS1 cells displayed a growth curve with a doubling time of about 3.7 ± 0.28, which is slower compared to the S-RMS cell line established by Yoshimatsu [12] and Schleicher [13], in keeping with the less aggressive clinical behavior. Furthermore, the S-RMS1 cell line displayed a weak ability to autocrine growth and form colonies in soft agar with an intermediate migration rate with respect to other two RMS cell lines: RH30 and RD18.

We found that S-RMS1 presented low levels of MEK-p and an absence of AKT-p and YAP-p proteins in comparison with RH30 and RD18. Cen and collaborators observed that AKT is frequently phosphorylated in ARMS and ERMS tissue microarray (TMA), indicating an activation of PDK-1/AKT pathway in this tumor, which plays a pivotal role in cell proliferation and survival [26]. In the S-RMS1, AKT is not phosphorylated, and this could also explain the low duplication rate of these cells. Our study reveals that YAP is not phosphorylated in the S-RMS cell line and that RD18 cells have a lower level of cytoplasmic YAPp in comparison to RH30 cells. Dysfunction or suppression of the Hippo pathway leads to a persistent activation of the unphosphorylated YAP, which often contributes to cancer development. Unphosphorylated YAP translocates to the nucleus, where it binds the TEA domain-containing family of transcription factors (TEAD), behaves as an oncogene, and activates target genes involved in cell proliferation, survival, and tumor growth [27]. An increased expression of YAP has been described in many human adult and pediatric cancers, and its expression is associated with an advanced clinical stage and short overall survival in different tumors [28]. Several studies have revealed that nuclear YAP is more prevalent in ERMS and SRMS than in ARMS, even if its role in RMS has not been clarified yet [29,30]. Furthermore, at a transcriptional level, S-RMS1 showed a higher level of the neovascularization markers endoglin [22] and GATA-6 [31] while TGFβ1R was downregulated. This finding may explain the morphological pattern of our tumor, which appears very vascularized with stag-horn aspects of the vessels, which are typical of neo-angiogenesis and not found in other RMS tumors. A sequencing analysis revealed that S-RMS1 cells retained original genomic variants as the tumor at diagnosis. Gene ontology (GO) terms and KEGG pathways analysis identified cancer, FoxO signaling, and cell cycle as the pathways with the highest number of mutated genes. Among these variants, *ATM*, *TERT*, *CDKN1C*, and *CREBBP* were identified in the heterozygous condition in the patient and resulted in segregation in one of the healthy parents. Activation of checkpoint arrest and homologous DNA repair are necessary for the maintenance of genomic integrity during DNA replication. Germline and somatic ATM mutations or deletions have been associated with the well-characterized ataxia telangiectasia syndrome, which manifests with an increased cancer predisposition [32], and they are commonly found in lymphoid malignancies, as well as in a variety of solid tumors [33]. An association of human RMS with deletion/mutation of ATM gene was reported in 2003 by Zhang and coworkers [34]. TERT is the catalytic protein subunit of telomerase, which functions to maintain chromosomal integrity and genome stability [35,36]. Genome-wide association studies have identified multiple variants at the TERT locus that are associated with telomere length and risk of several cancers [37,38,39]. CDKN1C, also called p57^kip2^, is a cyclin-dependent kinase inhibitor belonging to the Cip/Kip family whose alterations are related to Beckwith–Wiedemann syndrome. Accumulating evidence indicates that the p57Kip2 protein is frequently downregulated in different types of human epithelial and nonepithelial cancers as a consequence of genetic and epigenetic events [40]. Interestingly, CDKN1C seems to be regulated at the epigenetic level by enhancer of zeste homolog 2 (EZH2) [41], which catalyzes the addition of methyl groups to lysine 27 of the N-tail of histone H3 (H3K27me) and induces epigenetic modification involved in various differentiation processes. EZH2 has been involved in several cancers and in particular in the regulation of skeletal myogenesis and in RMS pathogenesis [42]. CREBBP is a ubiquitously expressed transcriptional coactivator and lysine acetyltransferase. It regulates transcription by serving as scaffolds that bridge sequence-specific DNA binding factors and the basal transcriptional machinery [43], and it also facilitates transcription through the acetylation of histones, transcription factors, and autoacetylation [44,45]. Even if the variants identified cannot be considered as certainly causative of the child’s disease, our results may provide an interesting base to further expand our knowledge on the pathogenic mechanisms of this rare RMS subtype.

In conclusion, we generated and characterized in vitro a new cell line, named S-RMS1, which is derived from an infantile spindle cell RMS tumor. To our knowledge, this is the first cell line established from this very rare pediatric tumor with myogenic characteristics and indolent behavior. The characterization of this cell line has further contributed to define the characteristics of the rare group of SRF-NCOA2 neoplasms and their rhabdomyoblastic nature, clearly demonstrated by MYOD1 and myogenin expression by tumor cells. Furthermore, it confirms the need for an accurate molecular–morphologic characterization of these tumors in clinical practice, in order to avoid the risk of underdiagnosis of these tumors as myofibroblastic lesions with SRF fusions [25].

## 4. Materials and Methods

### 4.1. Patient and Tumor Characteristics

A full-term newborn boy presented with a right flank mass showing progressive growth. Pregnancy and delivery were reported as uncomplicated. He had no congenital anomalies or dysmorphic signs. His family history for cancer was negative. Magnetic resonance imaging (MRI), which was performed at diagnosis, confirmed a lesion at the level of the right latissimus dorsi muscle. The patient underwent a needle biopsy of the lesion. A diagnosis of infantile spindle cell RMS was given, based on morphology and immunohistochemistry. Molecular studies by real time polymerase chain reaction (RT-PCR) revealed the SRF-NCOA2 fusion transcript. The child was classified as group III according to the Intergroup Rhabdomyosarcoma Study Group (IRSG) classification and stage II according to the TNM pretreatment staging classification [46]. According to the European Pediatric Soft Tissue Sarcoma Study Group (EpSSG) protocol (NCT#00339118), he received 3 courses of neoadjuvant chemotherapy with vincristine, actinomycin-D, and cyclophosphamide (age and weight adapted doses). Due to age, we decided to replace ifosfamide with cyclophosphamide for the first 3 cycles in order to reduce renal toxicity. Then, he underwent a macroscopically complete surgical excision of the mass, which appeared as an oval hard nodule of 3.8 × 3.5 × 1.5 cm^3^ of diameter. On microscopic examination, the diagnosis of infantile spindle cell RMS was confirmed. The tumor was almost completely vital (necrosis <1%, marginal excision). The tumor tissue obtained at this time of surgery was used to establish the cell line (S-RMS1). Then, the patient received 6 cycles of vincristine, actinomycin-D, and ifosfamide. Radiotherapy was not delivered, considering the young age (<1 year old). After 10 months from stop therapy, he presented a local relapse of tumor. He received 2 cycles of second line chemotherapy with vincristine, temozolomide, and irinotecan; a radiological disease reassessment showed stable disease. Thus, the patient underwent wide surgical resection of the lesion including part of right iliac wing, which was adherent to the mass. The histology showed a morphology similar to the original tumor, without increase of cytologic atypia or mitotic activity. Necrosis was absent. Surgical margins were infiltrated. Even this time, radiotherapy was not delivered considering the young age (2 years old) and the reported good prognosis of this tumor type [11]. Currently, the patient is doing well and is in complete clinical remission after 3 years and 6 months from the diagnosis. He is undergoing a strict radiological follow-up with whole body MRI every 3 months. Written informed consent for the use of clinical material for this study was obtained from the parents of the donor child.

### 4.2. Cell Line Establishment

The cell line was derived from the fresh tumor obtained from the surgery performed after 3 courses of neoadjuvant chemotherapy. This was established by mechanical disaggregation of the aseptic surgical sample, after three washes in saline solution. Small fragments of tissue were seeded in 24-well plates at high dilution. The culture medium used was DMEM low glucose (Euroclone) supplemented with 20% fetal bovine serum (FBS, Gibco), 2 mmol/L l-glutamine (Euroclone) and 100 g/mL penicillin-streptomycin (Euroclone). Cells were maintained at 37 °C in an atmosphere containing 5% CO_2_. When a confluence of spread cells from fragments of tissue was reached, cells were carefully detached and passed in new flasks. Cells were monitored daily to evaluate growth and morphology, and they were carefully detached and re-seeded upon reaching confluence. The RH30 cell line was maintained in RPMI 10% FBS, 2 mmol/L l-glutamine (Euroclone), and 100 g/mL penicillin-streptomycin (Euroclone), whereas the RD18 cell line was cultured in DMEM high glucose 2 mmol/L l-glutamine (Euroclone) and 100 g/mL penicillin-streptomycin (Euroclone). Cell line authentication was achieved by using short tandem repeat (STR) DNA fingerprinting (Eurofins Medigenomix, Ebersberg, Germany), and cells were regularly tested for mycoplasma-free infection.

### 4.3. Immunohistochemistry (IHC)

Representative sections from the formalin-fixed paraffin-embedded (FFPE) tumor sample were selected, and 2.5 μm thick serial sections were deparaffinized in xylene, rehydrated, and washed by using double distilled water. These sections were used for hematoxylin and eosin (H&E) and immunohistochemical staining for MyoD-1, myogenin, desmin, and SMA. Before the staining, the sections were pretreated with DAKO PT link (PT200) in high pH solution (cod K8004, DAKO North America, Carpinteria, CA, USA) for antigen retrieval. The immunostaining was done at 4 °C overnight using the following monoclonal mouse antihuman antibodies as the primary antibody: anti-MyoD-1 (Ready-to-Use, DAKO North America, CA), anti-myogenin (Ready-to-Use, DAKO North America, CA), anti-Desmin (IR606, Ready-to-Use, DAKO North America, CA), and anti-SMA (Ready-to-Use, DAKO North America, CA). En Vision Flex/HRP (cod K8024, Ready-to-Use, DAKO North America, CA) was used as a secondary antibody. The sections were then reacted in chromogen 3,3′-diaminobenzidine to detect the peroxidase activity, counterstained with hematoxylin, and mounted with cover slips.

### 4.4. Whole Genome Resequencing and Bioinformatic Analysis

DNA was extracted according to the MagPurix Tissue DNA Extraction Kit (Resnova, Rome, Italy) for an automatic extraction of genomic DNA. For each sample, the total amount of 1 μg of DNA based on the NanoDrop quantification method, with a 260/280 ratio >1.8, was used for whole genome sequencing analysis. Whole genome sequencing was performed at Macrogen Clinical Lab (https://www.macrogen.com, accessed on 15 December 2019) using an Illumina TruSeq DNA PCR Free library preparation kit. The DNA library was sequenced using an Illumina HiSeq X Ten sequencer. Sequence alignment on the hg19 sequence was performed with the Isaac aligner [47]. The Isaac variant caller was used to produce an annotated VCF, using the following reference variation databases: dbSNP138, dbSNP142, and 1000 Genomes phase I release v3. The resulting VCF file was uploaded to the Genoox analysis software (http://www.genoox.com, accessed on 17 December 2019) for variant prioritization. This software uses an innovative machine-learning approach to add an interpretative layer, based on a massive and automated integration of all the publicly available variation and mutation-to-disease publicly available resources to add a phenotype prioritization layer to the variant analysis (https://www.genoox.com/publications/, accessed on 18 December 2019). The most effective term for prioritization was indeed “rhabdomyosarcoma”, both for the tumoral sample and the cell line. The tumor sample and the cell line were analyzed separately and also intersected, which allowed for the identification of both the shared and unique variants. The list of prioritized genes of the shared variants was then used for a number of functional annotation analyses, moving from a gene-centered to a functional analysis network and finally a pathway-focused approach: Ensembl Variant Effect Predictor (https://www.ensembl.org/info/docs/tools/vep/index.html, accessed on 20 December 2019); DAVID (https://david.ncifcrf.gov/, accessed on 20 December 2019); Reactome database through ReactomeFiVis (https://reactome.org/tools/reactome-fiviz, accessed on 20 December 2019); and finally the KEGG Pathway Database through the Kegg Mapper software (https://www.kegg.jp/kegg/tool/map_pathway2.html, accessed on 20 December 2019). The FunRich software was finally used to integrate the bioinformatic analysis (http://www.funrich.org/, accessed on 20 December 2019). The bioinformatic analysis was performed in collaboration with Nico Innovagroup (https://nicoinnovagroup.com/en/index.html, accessed on 20 December 2019). The NGS whole genome sequences both for the spindle cell rhabdomyosarcoma tumor sample and the S-RMS1 cell line were uploaded and registered with the European Bioinformatics Institute’s European Nucleotide Archive (EBI/ENA: https://www.ebi.ac.uk/ena, accessed on 20 December 2019) in BAM file format (alignment on the homo sapiens hg19 reference in binary format), as required by EBI/ENA specifications. The accession number for the study, including the two NGS datasets, is PRJEB38125. The accession number for the tumor sample is ERS4540366, while for the cell line it is ERS4540365. These datasets are confidential until public release of the results of the associated scientific project.

### 4.5. Clinical Exome Sequencing of Genomic DNA and Germline Variant Identification

After obtaining informed consent for genetic testing, molecular characterization of blood DNA in the patient and his parents was performed by next generation sequencing (NGS), using clinical exome sequencing (CES) with a Twist Human Core Exome Kit (Twist Bioscience). The BaseSpace pipeline (Illumina, https://basespace.illumina.com) and the TGex software (LifeMap Sciences, Inc., Walnut, CA, USA) were used for the variant calling and annotating variants, respectively. Variants identified as pathogenic were visualized by the Integrative Genome Viewer (IGV). Paired-end reads of 101bp were generated, with a mean coverage of 60 to 96×. The BWA alignment algorithm was used to map sequence reads to the UCSC human genome reference build 19. Variants altering the coding sequence were selected that were present at a frequency of <1:100 (0.01) in the control population, and any that were present in GnomAD with a Minor Allele Frequency MAF ≥ 0.01 were excluded. Targeted cancer-related genes were selected for analysis, on the basis of the American College of Medical Genetics and Genomics (ACMG) gene list [48], the Online Mendelian Inheritance in Man (OMIM) [49], the LOVD database [50] and the literature.

### 4.6. DNA Methylation Profiling

DNA methylation profiling was performed according to protocols approved by the institutional review board. Samples were analyzed using Illumina Infinium Human MethylationEPIC BeadChip (EPIC) arrays (Illumina, San Diego, CA, USA) according to the manufacturer’s instructions, on the Illumina iScan Platform (Illumina, San Diego, USA), as previously reported [51]. In detail, 250 or 500 ng DNA was used as input material from FFPE or fresh frozen cells, respectively. EPIC BeadChip data were analyzed as previously reported [51,52] by means of R (V.3.6.1), using the ChAMP package (V.2.16.1) for quality checks and filters, to calculate methylation levels and to functionally annotate probes at the gene level. Multidimensional scaling (MDS) on the cohort samples was performed using the cmdscale function, with Euclidean distance. A cohort of 5 ERMS diagnosed at Bambino Gesù Children’s Hospital was used as the control group. Demographic and clinical features of samples included in DNA methylation profiling are reported in Table 4.

### 4.7. RNA-Extraction, RT-PCR, and RT-qPCR

The Trizol Reagent (Invitrogen, Milan, Italy) was used to extract total RNA from cells at III and VII passages according to the manufacturer’s instructions. One microgram of total RNA was reverse-transcribed by using the SuperScript II Reverse Transcriptase (Invitrogen, Milan, Italy). PCR amplification was performed by using the BIOTAQ DNA Polymerase (Bioline, London, UK) according to the manufacturer’s instructions. The PCR reaction mixture contained 1.5 mM MgCl_2_, 0.2 µM of each primer, 1 × PCR Buffer, 0.4 mM of each dNTPs, 0.5 U of Taq polymerase, and 1 µL of the RT product in a final 20 µL reaction volume. SRF-NCOA2 transcript was amplified using primer pairs: SRF_for 5′-TTCCTGACAGCATCATCTGGG-3′ and NCOA2_rev 5′-AATCTCCTCCAAGTTGTCCAGC-3′, Myod-1_for 5′-AGCACTACAGCGGCGACT-3′ rev 5′-GCGACTCAGAAGGCACGTC-3′, Myogenin_for 5′-TAAGGTGTGTAAGAGGAAGTC-3′ rev_ 5′-TACATGGATGAGGAAGGGGAT-3′, ß2-microglobulin_for5′-GTGGAGCATTCAGACTTGTCTTTCAGCA-3′ rev_5′-TTCATCCAATCCAAATGCGGCATCTTC-3′. PCR conditions were performed with the following cycle profile: 94 °C for 2 min, followed by 40 cycles of 94 °C for 15 s, 60 °C for 15 s, and 72 °C for 15 s. After the last cycle, an extended 10 min at 72 °C was followed by cooling to 4 °C. ß2-microglobulin expression was concomitantly assessed as a control for the presence of amplifiable RNA and for efficiency of reverse transcription. PCR reaction products were electrophoresed through 3% agarose gels, and their sizes were determined by a comparative analysis with DNA Marker Ladder 50 (Invitrogen, Milan, Italy). TaqMan gene assay (Applied Biosystems, Life Technologies, Carlsbad, CA, USA) for endoglin (Hs00923996_m1), TGFbetaR1 (Hs00610320_m1), MET (Hs01565584_m1), CX3CL1 (Hs00171086_m1), myogenin (Hs01072232_m1), Mef2A (Hs01050406_g1), Mef2B (Hs04188747_m1), Mef2C (Hs00231149_m1), and Mef2D (Hs00954735_m1) were used for the relative quantification of the gene expression by RT-qPCR. The samples were normalized according to the glyceraldehyde-3-phosphate dehydrogenase (GAPDH) mRNA (Hs99999905_m1) levels. An Applied Biosystems 7900HT Fast RealTime PCR System (Applied Biosystems) was used for the measurements. The expression fold change was calculated by the 2^−ΔΔCt^ method for each of the reference genes [53]. At least two independent amplifications were performed for each probe, with triplicate samples.

### 4.8. Western Blot

Protein extraction was performed with 1X Cell Lysis Buffer (20 mM Tris (pH 7.5), 150 mM NaCl, 1 mM EDTA, 1 mM EGTA, 1% Triton X-100, 2.5 mM sodium pyrophosphate, 1 mM β-glycerophosphate, 1 mM Na3VO4, 1 μg/mL leupeptin, 1mM PMSF) (#9803 Cell Signaling) lysates were incubated on ice for 30 min and centrifugated at 12,000 g for 20 min at 4 °C. Equal micrograms (20 μg) of proteins quantified with BCA and boiled in an SDS sample buffer (200 mM Tris–HCl (pH 6.8)), 40% glycerol, 20% β-mercaptoethanol, 4% sodium dodecyl sulfate, and bromophenol blue) were resolved on 10% SDS-PAGE and transferred to Immun-Blot^®^ PVDF membranes (Bio-Rad, Hercules, California, USA). Blots were blocked for 1 h in TBS-T (TBS plus 0.05% Tween-20), 5% nonfat, dried milk and probed overnight at 4 °C with anti-phospho Erk 1/2 (#9102 Cell Signaling Technology^®^),anti-Erk 1/2 (#9101 Cell Signaling Technology^®^, Danvers, MA, USA), anti-phospho-Akt (Ser473) (#9271 Cell Signaling Technology^®^), anti-Akt (# 9272 Cell Signaling Technology^®^), anti-Phospho YAP1 (ab76252 Abcam), anti-YAP 1 (ab52771 Abcam), anti-phospho MEK 1/2 (Ser217/221) (#9121 Cell Signaling Technology^®^), anti-Phospho-mTOR (Ser2448) (#2971 Cell Signaling Technology^®^), anti-mTOR (#2972 Cell Signaling Technology^®^), and anti-GAPDH (D16H11) XP (#5174 Cell Signaling Technology^®^). Immunocomplexes were detected with horseradish peroxidase-conjugated species-specific secondary antibodies (Santa Cruz Biotechnology, Dallas, TX, USA) followed by an enhanced chemiluminescence reaction with ECL Plus Detection Reagents (Amersham).

### 4.9. Growth Characteristics

To determine cell growth, 2 × 10^5^ cells were seeded onto 12-well plates, and the average number of cells in triplicate dishes was counted each day for one week. Cells were harvested by trypsinization and counted with the Bürker counting chamber and the trypan blue dye exclusion method. The doubling time was obtained from the time necessary for the cell population in the logarithmic phase to double. The growth curve was compared to that of RH30 and RD18 cell lines. The number of cells was expressed as mean ± SEM, and the experiment was repeated at least three times for each cell line considered.

### 4.10. Serum Independent Growth

Single cell suspension (1 × 10^4^ cell/well) of S-RMS1 and RD18 cells were plated into 24-well plates at different culture conditions of DMEM media supplemented with 20% FBS, DMEM media supplemented with 10% FBS, DMEM media supplemented with 5% FBS, and DMEM without serum. In the same way, 1 × 10^4^ cell/well of RH30 cells were plated and maintained in RPMI media supplemented with 20% FBS, RPMI media supplemented with 10% FBS, RPMI media supplemented with 5% FBS, and RPMI without serum. Cultures were maintained in the incubator at 37 °C in 5% CO_2_ for 6 days and examined by microscopy every day, while the morphology of the cells was inspected continuously. The media were changed every 2 days; the cells were detached by trypsinization every 24 h, and the cells’ numbers were counted using a Bürker counting chamber and trypan blue dye exclusion method. All counts were performed in triplicate, and three independent experiments were performed.

### 4.11. Wound Healing Assay

Migration was performed with an Ibidi culture-insert (Ibidi^®^) as per the manufacturer’s instructions. Briefly, cell suspensions of Cat-1, RH30, and RD18 cells were prepared (3–4 × 10^5^ cells/mL), and 70 μL were applied into each well. Cells were incubated at 37 °C and 5% CO2 for 24 h. After appropriate cell attachment, culture-inserts were gently removed, after which a fresh medium was added, and images were captured immediately (day 0) and also at 24 and 36 h later with a Leica DMi8 Inverted Microscope. Cell migration was quantitatively assessed, measuring the entire area of the scratches by ImageJ software (Wayne Rasband, NIH, Bethesda, MD, USA). The results were obtained from measurements of the total area of the scratch between the wound edges per scratch from two separate experiments for each cell line, expressed as a fold change over either control ones.

### 4.12. Soft Agar Colony Formation Assay

S-RMS1 cells were assayed for their capacity to form colonies in soft agar. RH30 and RD18 cells were used in the same condition for comparison. A total of 1 × 10^3^ cells were suspended in DMEM (10% FBS) or RPMI (10% FBS) containing 0.35% agar (Difco™ Agar Noble BD). Cells were seeded on a layer of 0.7% agar in DMEM (10% FBS) or RPMI (10% FBS) in 6 multi-well plates. Medium was refreshed every 2 days. On week 4, colonies were counted by microscopic inspection at a magnification of 10×. Triplicate assays were carried out in three independent experiments.

### 4.13. Statistical Analysis

Experiments were performed in at least three independent repeats. Student’s two-tailed *t*-test was used to evaluate the statistical significance of the data; *p*-value of <0.05 was considered as being statistically significant. For the variant prioritization, Genoox confidence calls together with a global quality score and effect prediction of the variants were considered for the identification of the gene list. For all the ensuing bioinformatic analyses focused on functional annotation, the values of the standard statistical indicators (*p* value and FDR) were used to assess significance.

For the variants analysis on genomic DNA, TGex software uses the Genome Analysis Toolkit [54] to calculate the allelic frequency of variants and a genotype-based likelihood ratio test (LRT) to compute the *p*-value of associations [55]. A design of association studies with pooled or unpooled next-generation sequencing data can be considered.

## Figures and Tables

**Table 1:** List of genes presenting variants (SNPs and indels) only in tumor at diagnosis.

Gene	Variation Type	Chr	dbSNP	AA Change	Zygosity	Region	Effect
*TBP*	Indel	chr6	rs1478666781	p.Gln73_Gln78del	het	Exonic	Nonframeshift
*CASQ2*	Indel	chr1	rs1491387135		het	Splice Region	
*IL23R*	Indel	chr1	rs779016240		hom	Splice Region	
*FCGR2A*	SNP	chr1	rs382627	p.Leu273Pro	het	Exonic	Missense

Chr: chromosome; AA, aminoacid; het: heterozygote; hom: homozygote.

**Table 2:** List of genes presenting variants (SNPs and indels) only in S-RMS1 cell line.

Gene	Variation Type	Chr	dbSNP	AA Change	Zygosity	Region	Effect
*LRRC37B*	SNP	chr17	rs471887	p.Gln621His	het	Exonic	Missense
*TPTE2*	SNP	chr13	rs78472618	p.Lys39Glu	het	Exonic	Missense

Chr: chromosome; AA, aminoacid; het: heterozygote; hom: homozygote.

**Table 3:** List of genes presenting germline variants in patient’s gDNA.

Gene	Variation Type AA Change	Location	dbSNP	ACMG	Segregation	MAX AF (%)	SIFT–PolyPhen
POLE	c.5221C>T p.Gln1741*	12:133218390	rs781481160	LP	Mat	0.00082	
CDKN1C	c.392_394delAGG p.Glu131del	11:2906325		VUS	Pat	0	
TERT	c.922C>A p.Pro308Thr	5:1294079		VUS	Mat	0	
ATM	c.8428A>C p.Lys2810Gln	11:108216479	rs730881325	VUS	Mat	0.004	0.05–0.04
CREBBP	c.5800T>C p.Ser1934Pro	16:3779248	rs587783504	VUS	Mat	0.035	0.035

Note: List of genes and germline variants studied on gDNA in the NGS analysis, associated with clinical patient features. Exome sequencing filtering and prioritization identified five potential candidate variants that were absent or present with an MAF < 0.01 in population databases. Variants were subsequently ranked by their potential functional impact using PolyPhen and SIFT. Gene reference sequences utilized were NM_006231.3 (*POLE*), NM_000076.2 (*CDKN1C*), NM_198253.2 (*TERT*), NM_000051.3 (*ATM*), and NM_004380.2 (*CREBBP*). All variants were identified in the heterozygous condition in patient. The frequency distributions of the previously described variants are consistent with the frequency distributions observed in the general population. In the table, the genomic locations, the prediction analysis, and American College of Medical Genetics (ACMG) classification of the variants in the genes are reported.

**Table 4:** Demographic and clinical features of samples included in DNA methylation profiling.

Sample	Sex	Age (Months)	Histology	Specimen	Location
S-RMS1	Male	2	SS-RMS	Tumor_cells	Right dorsal muscle
S-RMS1-T	Male	2	SS-RMS	Tumor_FF	Right dorsal muscle
E-RMS_2	Female	14	E-RMS	Tumor_FFPE	Right psoas muscle
E-RMS_3	Male	5	E-RMS	Tumor_FFPE	abdomen
E-RMS_4	Male	240	E-RMS	Tumor_FFPE	Left paratesticular
E-RMS_5	Female	29	E-RMS botryoid variant	Tumor_FFPE	Bladder
E-RMS_6	Male	84	E-RMS	Tumor_FFPE	Prostate

RMS: rhabdomyosarcoma; E: embryonal; SS: spindle and sclerosing; FF: fresh frozen; FFPE: formalin-fixed paraffin-embedded.

## Data Availability

The data presented in this study are openly available in European Nucleotide Archive (ENA) at https://www.ebi.ac.uk/ena/browser/home. The accession number for the study, including the two NGS datasets, is PRJEB38125. The accession number for the tumor sample is ERS4540366, while for the cell line it is ERS4540365.

## Supplementary Materials

The following are available online at https://www.mdpi.com/article/10.3390/ijms22115484/s1. Figures S1–S4; Table S1. List of shared genes presenting variants (SNPs and INDELs) both in tumor tissue and in the S-RMS1 cell line.

## References

[1] Russo I., Di Paolo V., Gurnari C., Mastronuzzi A., Del Bufalo F., Di Paolo P.L., Di Giannatale A., Boldrini R., Milano G.M. (2018). Congenital Rhabdomyosarcoma: A different clinical presentation in two cases. BMC Pediatr..

[2] Missiaglia E., Williamson D., Chisholm J., Wirapati P., Pierron G., Petel F., Concordet J.P., Thway K., Oberlin O., Pritchard-Jones K. (2012). PAX3/FOXO1 fusion gene status is the key prognostic molecular marker in rhabdomyosarcoma and significantly improves current risk stratification. J. Clin. Oncol..

[3] Cavazzana A.O., Schmidt D., Ninfo V., Harms D., Tollot M., Carli M., Treuner J., Betto R., Salviati G. (1992). Spindle cell rhabdomyosarcoma. A prognostically favorable variant of rhabdomyosarcoma. Am. J. Surg. Pathol..

[4] Leuschner I., Newton W.A., Schmidt D., Sachs N., Asmar L., Hamoudi A., Harms D., Maurer H.M. (1993). Spindle cell variants of embryonal rhabdomyosarcoma in the paratesticular region. A report of the Intergroup Rhabdomyosarcoma Study. Am. J. Surg. Pathol..

[5] Mentzel T., Kuhnen C. (2006). Spindle cell rhabdomyosarcoma in adults: Clinicopathological and immunohistochemical analysis of seven new cases. Virchows Arch..

[6] Nascimento A.F., Fletcher C.D. (2005). Spindle cell rhabdomyosarcoma in adults. Am. J. Surg. Pathol..

[7] Mentzel T., Katenkamp D. (2000). Sclerosing, pseudovascular rhabdomyosarcoma in adults. Clinicopathological and immunohistochemical analysis of three cases. Virchows Arch..

[8] Fletcher C.D.M., Bridge J.A., Hogendoorn P.C.W. (2013). WHO Classification of Tumours of Soft Tissue and Bone.

[9] Lundgren L., Angervall L., Stenman G., Kindblom L.G. (1993). Infantile rhabdomyofibrosarcoma: A high-grade sarcoma distinguishable from infantile fibrosarcoma and rhabdomyosarcoma. Hum. Pathol..

[10] Mosquera J.M., Sboner A., Zhang L., Kitabayashi N., Chen C.L., Sung Y.S., Wexler L.H., LaQuaglia M.P., Edelman M., Sreekantaiah C. (2013). Recurrent NCOA2 gene rearrangements in congenital/infantile spindle cell rhabdomyosarcoma. Genes Chromosomes Cancer.

[11] Alaggio R., Zhang L., Sung Y.S., Huang S.C., Chen C.L., Bisogno G., Zin A., Agaram N.P., LaQuaglia M.P., Wexler L.H. (2016). A Molecular Study of Pediatric Spindle and Sclerosing Rhabdomyosarcoma: Identification of Novel and Recurrent VGLL2-related Fusions in Infantile Cases. Am. J. Surg. Pathol..

[12] Yoshimatsu Y., Noguchi R., Tsuchiya R., Sei A., Sugaya J., Iwata S., Sugiyama M., Yoshida A., Kawai A., Kondo T. (2020). Establishment and characterization of NCC-ssRMS1-C1: A novel patient-derived spindle-cell/sclerosing rhabdomyosarcoma cell line. Hum. Cell.

[13] Schleicher S., Grote S., Malenke E., Chan K.C., Schaller M., Fehrenbacher B., Riester R., Kluba T., Frauenfeld L., Boesmueller H. (2020). Establishment and Characterization of a Sclerosing Spindle Cell Rhabdomyosarcoma Cell Line with a Complex Genomic Profile. Cells.

[14] Tsuchiya S., Yoshimatsu Y., Noguchi R., Ono T., Sei A., Takeshita F., Sugaya J., Fukushima S., Yoshida A., Ohtori S. (2021). Establishment and characterization of NCC-DDLPS3-C1: A novel patient-derived cell line of dedifferentiated liposarcoma. Hum. Cell.

[15] De Vita A., Recine F., Mercatali L., Miserocchi G., Liverani C., Spadazzi C., Casadei R., Bongiovanni A., Pieri F., Riva N. (2017). Myxofibrosarcoma primary cultures: Molecular and pharmacological profile. Ther. Adv. Med. Oncol..

[16] Miserocchi G., Mercatali L., Liverani C., De Vita A., Spadazzi C., Pieri F., Bongiovanni A., Recine F., Amadori D., Ibrahim T. (2017). Management and potentialities of primary cancer cultures in preclinical and translational studies. J. Transl. Med..

[17] Racanelli D., Brenca M., Baldazzi D., Goeman F., Casini B., De Angelis B., Guercio M., Milano G.M., Tamborini E., Busico A. (2020). Next-Generation Sequencing Approaches for the Identification of Pathognomonic Fusion Transcripts in Sarcomas: The Experience of the Italian ACC Sarcoma Working Group. Front. Oncol..

[18] Xu J., O’Malley B.W. (2002). Molecular mechanisms and cellular biology of the steroid receptor coactivator (SRC) family in steroid receptor function. Rev. Endocr. Metab. Disord..

[19] Pipes G.C., Creemers E.E., Olson E.N. (2006). The myocardin family of transcriptional coactivators: Versatile regulators of cell growth, migration and myogenesis. Genes Dev..

[20] Tsaousis G.N., Papadopoulou E., Apessos A., Agiannitopoulos K., Pepe G., Kampouri S., Diamantopoulos N., Floros T., Iosifidou R., Katopodi O. (2019). Analysis of hereditary cancer syndromes by using a panel of genes: Novel and multiple pathogenic mutations. BMC Cancer.

[21] Koelsche C., Schrimpf D., Stichel D., Sill M., Sahm F., Reuss D., Blattner M., Worst B., Heilig C.E., Bec K. (2021). Sarcoma classification by DNA methylation profiling. Nat. Commun..

[22] Di Paolo V., Russo I., Boldrini R., Ravà L., Pezzullo M., Benedetti M.C., Galardi A., Colletti M., Rota R., Orlando D. (2018). Evaluation of Endoglin (CD105) expression in pediatric rhabdomyosarcoma. BMC Cancer.

[23] Jo V.Y., Fletcher C.D.M. (2014). WHO classification of soft tissue tumours: An update based on the 2013 (4th) edition. Pathology.

[24] Butel T., Marie Karanian M., Pierron G., Orbach D., Ranchere D., Cozic N., Louise Galmiche L., Coulomb A., Corradini N., Lacour B. (2020). Integrative clinical and biopathology analyses to understand the clinical heterogeneity of infantile rhabdomyosarcoma: A report from the French MMT committee. Cancer Med..

[25] Karanian M., Kelsey A., Paindavoine S., Duc A., Vanacker H., Hook L., Weinbreck N., Delfour C., Minard V., Baillard P. (2020). SRF Fusions Other Than With RELA Expand the Molecular Definition of SRF-fused Perivascular Tumors. Am. J. Surg. Pathol..

[26] Cen L., Hsieh F.C., Lin H.J., Chen C.S., Qualman S.J., Lin J. (2007). PDK-1/AKT pathway as a novel therapeutic target in rhabdomyosarcoma cells using OSU-03012 compound. Br. J. Cancer.

[27] Pfleger C.M. (2017). The Hippo pathway: A master regulatory network important in development and dysregulated in disease. Curr. Top. Dev. Biol..

[28] Mohamed A.D., Tremblay A.M., Murray G.I., Wackerhage H. (2015). The Hippo signal transduction pathway in soft tissue sarcomas. Biochim. Biophys. Acta.

[29] Ahmed A.A., Habeebu S.S., Sherman A.K., Ye S.Q., Wood N., Chastain K.M., Tsokos M.G. (2018). Potential Value of YAP Staining in Rhabdomyosarcoma. J. Histochem. Cytochem..

[30] Tremblay A.M., Missiaglia E., Galli G.G., Hettmer S., Urcia R., Carrara M., Judson R.N., Thway K., Nadal G., Selfe J.L. (2014). The Hippo transducer YAP1 transforms activated satellite cells and is a potent effector of embryonal rhabdomyosarcoma formation. Cancer Cell.

[31] Froese N., Kattih B., Breitbart A., Grund A., Geffers R., Molkentin J.D., Kispert A., Wollert K.C., Drexler H., Heineke J. (2011). GATA6 promotes angiogenic function and survival in endothelial cells by suppression of autocrine transforming growth factor beta/activin receptor-like kinase 5 signaling. J. Biol. Chem..

[32] Peterson R.D., Funkhouser J.D., Tuck-Muller C.M., Gatti R.A. (1992). Cancer susceptibility in ataxia-telangiectasia. Leukemia.

[33] Reiman A., Srinivasan V., Barone G., Last J.I., Wootton L.L., Davies E.G., Verhagen M.M., Willemsen M.A., Weemaes C.M., Byrd P.J. (2011). Lymphoid tumours and breast cancer in ataxia telangiectasia; substantial protective effect of residual ATM kinase activity against childhood tumours. Br. J. Cancer.

[34] Zhang P., Bhakta K.S., Puri P.L., Newbury R.O., Feramisco J.R., Wang J.Y. (2003). Association of ataxia telangiectasia mutated (ATM) gene mutation/deletion with rhabdomyosarcoma. Cancer Biol. Ther..

[35] Greider C.W., Blackburn E.H. (2004). Tracking telomerase. Cell.

[36] Blasco M.A. (2005). Telomeres and human disease: Ageing, cancer and beyond. Nat. Rev. Genet..

[37] Bojesen S.E., Pooley K.A., Johnatty S.E., Beesley J., Michailidou K., Tyrer J.P., Edwards S.L., Pickett H.A., Shen H.C., Smart C.E. (2013). Multiple independent variants at the TERT locus are associated with telomere length and risks of breast and ovarian cancer. Nat. Genet..

[38] Codd V., Nelson C.P., Albrecht E., Mangino M., Deelen J., Buxton J.L., Hottenga J.J., Fischer K., Esko T., Surakka I. (2013). Identification of seven loci affecting mean telomere length and their association with disease. Nat. Genet..

[39] Rafnar T., Sulem P., Stacey S.N., Geller F., Gudmundsson J., Sigurdsson A., Jakobsdottir M., Helgadottir H., Thorlacius S., Aben K.K. (2009). Sequence variants at the TERT-CLPTM1L locus associate with many cancer types. Nat. Genet..

[40] Borriello A., Caldarelli I., Bencivenga D., Criscuolo M., Cucciolla V., Tramontano A., Oliva A., Perrotta S., Della Ragione F. (2011). p57(Kip2) and cancer: Time for a critical appraisal. Mol. Cancer Res..

[41] Yang X., Karuturi R.K.M., Sun F., Aau M., Yu K., Shao R., Miller L.D., Tan P.B.O., Yu Q. (2009). CDKN1C (p57) is a direct target of EZH2 and suppressed by multiple epigenetic mechanisms in breast cancer cells. PLoS ONE.

[42] Marchesi I., Giordano A., Bagella L. (2014). Roles of enhancer of zeste homolog 2: From skeletal muscle differentiation to rhabdomyosarcoma carcinogenesis. Cell Cycle.

[43] Chan H.M., La Thangue N.B. (2001). p300/CBP proteins: HATs for transcriptional bridges and scaffolds. J. Cell Sci..

[44] Sterner D.E., Berger S.L. (2000). Acetylation of histones and transcription-related factors. Microbiol. Mol. Biol. Rev..

[45] Black J.C., Choi J.E., Lombardo S.R., Carey M. (2006). A mechanism for coordinating chromatin modification and preinitiation complex assembly. Mol. Cell.

[46] Lawrence W., Anderson J.R., Gehan E.A., Maurer H. (1997). Pretreatment TNM staging of childhood rhabdomyosarcoma: A report of the intergroup rhabdomyosarcoma study group. Children’s cancer study group. Pediatric oncology group. Cancer.

[47] Raczy C., Petrovski R., Saunders C.T., Chorny I., Kruglyak S., Margulies E.H., Chuang H.Y., Kallberg M., Kumar S.A., Liao A. (2013). Isaac: Ultra-fast whole-genome secondary analysis on Illumina sequencing platforms. Bioinformatics.

[48] Green R.C., Berg J.S., Grody W.W., Kalia S.S., Korf B.R., Martin C.L., McGuire A.L., Nussbaum R.L., O’Daniel J.M., Ormond K.E. (2013). ACMG recommendations for reporting of incidental findings in clinical exome and genome sequencing. American College of Medical Genetics and Genomics. Genet. Med..

[49] McKusick-Nathans Institute of Genetic Medicine JHUB, MD. Online Mendelian Inheritance in Man, OMIM® 2016 [cited 2016]. http://omim.org/.

[50] Fokkema I.F., Taschner P.E., Schaafsma G.C., Celli J., Laros J.F., den Dunnen J.T. (2011). LOVD v.2.0: The next generation in gene variant databases. Hum. Mutat..

[51] Miele E., De Vito R., Ciolfi A., Pedace L., Russo I., De Pasquale M.D., Di Giannatale A., Crocoli A., De Angelis B., Tartaglia M. (2020). DNA Methylation Profiling for Diagnosing Undifferentiated Sarcoma with Capicua Transcriptional Receptor (CIC) Alterations. Int. J. Mol. Sci..

[52] Ballabio C., Anderle M., Gianesello M., Lago C., Miele E., Cardano M., Aiello G., Piazza S., Caron D., Gianno F. (2020). Modeling medulloblastoma in vivo and with human cerebellar organoids. Nat. Commun..

[53] Livak K.J., Schmittgen T.D. (2001). Analysis of relative gene expression data using real-time quantitative PCR and the 2(−Delta Delta C(T)) method. Methods.

[54] DePristo M.A., Banks E., Poplin R., Garimella K.V., Maguire J.R., Hartl C., Philippakis A.A., del Angel G., Rivas M.A., Hanna M. (2011). A framework for variation discovery and genotyping using next-generation DNA sequencing data. Nat. Genet..

[55] Kim S.Y., Li Y., Guo Y., Li R., Holmkvist J., Hansen T., Pedersen O., Wang J., Nielsen R. (2010). Design of association studies with pooled or un-pooled next-generation sequencing data. Genet. Epidemiol..

