# Supplementary material for: Establishment and Characterization of a Cell Line (S-RMS1) Derived from an Infantile Spindle Cell Rhabdomyosarcoma with SRF-NCOA2 Fusion Transcript
Source: Int J Mol Sci. 2021 May 22;22(11):5484. doi: 10.3390/ijms22115484 (PMC8196948; doi:10.3390/ijms22115484)
Supplement: Supplementary file 1 [file ijms-22-05484-s001.zip › final - Supplementary figure 24-05-2021 tif.pdf]

A

>sp|P11831|**SRF**_HUMAN Serum response factor
MLPTQAGAAAALGRGSALGGSLNRTPTGRPGGGGGTRGANGGRVPGNGAGLGPGRLEREA
AAAAATTPAPTAGALYSGSEGDSESGEEEELGAERRGLKRSLSEMEIGMVVGGPEASAAA
TGGYGPVSGAVSGAKPGKKTRGRVKIKMEFIDNKLRRYTTFSKRKTGIMKKAYELSTLTG
TQVLLLVASETGHVYTFATRKLQPMITSETGKALIQTCLNSPDSPPRSDPTTDQRMSATG
FEETDLTYQVSESDSSGETKDTLKPAFTVTNLPGTTSTIQTAPSTSTTMQVSSGPSFPIT
NYLAPVSASVSPSAVSSANGTVLKSTGSGPVSSGGLMQLPTSFTLMPGGAVAQQVPVQAI
QVHQAPQQASPSRDSSTDLTQTSSSGTVTLPATIMTSSVPTTVGGHMMYPSPHAVMYAPT
SGLGDGSLTVLNAFSQAPSTMQVSHSQVQEPGGVPQVFLTASSGTVQIPVSAVQLHQMAV
IGQQAGSSSNLTELQVVNLDTAHSTKSE

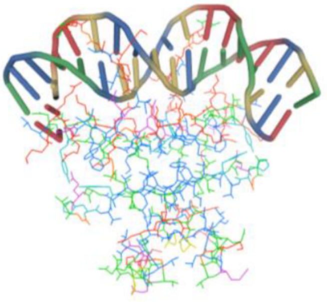

B

>sp|Q15596|**NCOA2**_HUMAN Nuclear receptor coactivator 2
MSGMGENTSDPSRAETRKRKECPDQLGPSPKRNTEKRNREQENKYIEELAELIFANFNDI
DNFNFKPDKCAILKETVKQIRQIKEQEKAAAANIDEVQKSDVSSTGQGVIDKDALGPMML
EALDGFFFVVNLEGNVVFVSENVTQYLRYNQEELMNKSVYSILHVGDHTEFVKNLLPKSI
VNGGSWSGEPPRRNSHTFNCRMLVKPLPDSEEEGHDNQEAHQKYETMQCFAVSQPKSIKE
EGEDLQSCLICVARRVPMKERPVLPSSESFTTRQDLQGKITSLDTSTMRAAMKPGWEDLV
RRCIQKFHAQHEGESVSYAKRHHHEVLRQGLAFSQIYRFSLSDGTLVAAQTKSKLIRSQT
TNEPQLVISLHMLHREQNVCVMNPDLTGQTMGKPLNPISSNSPAHQALCSGNPGQDMTLS
SNINFPINGPKEQMGMPMGRFGGSGGMNHVSGMQATTPQGSNYALKMNSPSQSSPGMNPG
QPTSMLSPRHRMSPGVAGSPRIPPSQFSPAGSLHSPVGVCSSTGNSHSYTNSSLNALQAL
SEGHGVSLGSSLASPDLKMGNLQNSPVNMNPPPLSKMGSLDSKDCFGLYGEPSEGTTGQA
ESSCHPGEQKETNDPNLPPAVSSERADGQSRLHDSKGQTKLLQLLTTKSDQMEPSPLASS
LSDTNKDSTGSLPGSGSTHGTSLKEKHKILHRLLQDSSSPVDLAKLTAEATGKDLSQESS
STAPGSEVTIKQEPVSPKKKENALLRYLLDKDDTKDIGLPEITPKLERLDSKTDPASNTK
LIAMKTEKEEMSFEPGDQPGSELDNLEEILDDLQNSQLPQLFPDTRPGAPAGSVDKQAII
NDLMQLTAENSPVTPVGAQKTALRISQSTFNNPRPGQLGRLLPNQNLPLDITLQSPTGAG
PFPPIRNSSPYSVIPQPGMMGNQGMIGNQGNLGNSSTGMIGNSASRPTMPSGEWAPQSSA
VRVTCAATTSAMNRPVQGGMIRNPAASIPMRPSSQPGQRQTLQSQVMNIGPSELEMNMGG
PQYSQQQAPPNQTAPWPESILPIDQASFASQNRQPFGSSPDDLLCPHPAAESPSDEGALL
DQLYLALRNFDGLEEIDRALGIPELVSQSQAVDPEQFSSQDSNIMLEQKAPVFPQQYASQ
AQMAQGSYSPMQDPNFHTMGQRPSYATLRMQPRPGLRPTGLVQNQPNQLRLQLQHRLQAQ
QNRQPLMNQISNVSNVNLTLRPGVPTQAPINAQMLAQRQREILNQHLRQRQMHQQQQVQQ
RTLMMRGQGLNMTPSMVAPSGMPATMSNPRIPQANAQQFPFPPNYGISQQPDPGFTGATT
PQSPLMSPRMAHTQSPMMQQSQANPAYQAPSDINGWAQGNMGGNSMFSQQSPPHFGQQAN
TSMYSNNMNINVSMATNTGGMSSMNQMTGQISMTSVTSVPTSGLSSMGPEQVNDPALRGG
NLFPNQLPGMDMIKQEGDTTRKYC

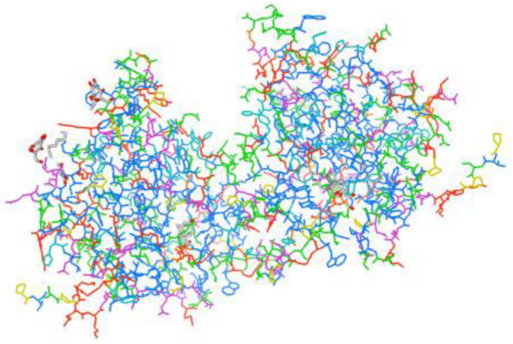

C

SRF-NCOA2
MLPTQAGAAAALGRGSALGGSLNRTPTGRPGGGGGTRGANGGRVPGNGAGLGPGRLEREA
AAAAATTPAPTAGALYSGSEGDSESGEEEELGAERRGLKRSLSEMEIGMVVGGPEASAAA
TGGYGPVSGAVSGAKPGKKTRGRVKIKMEFIDNKLRRYTTFSKRKTGIMKKAYELSTLTG
TQVLLLVASETGHVYTFATRKLQPMITSETGKALIQTCLNSPDSPPRSDPTTDQRMSATG
FEETDLTYQVSESDSSGETKDTLKPAFTVTNLPGTTSTIQTAPSTSTTMQVSSGPSFPIT
NYLAPVSASVSPSAVSSANGTVLKSTGSGPVSSGGLMQLPTSFTLMPGGAVAQQVPVQAI
QVHQAPQQASPSRDSSTDLTQTSSSGTVTLPATIMTSSVPTTVGGHMMYPSPHAVMYAPT
SGLGDGSLTVLNAFSQAPSTMQVSHSQVQEPGGVPQVFLTASSGTVQIPVSAVQLHQPGS
ELDNLEEILDDLQNSQLPQLFPDTRPGAPAGSVDKQAIINDLMQLTAENSPVTPVGAQKT
ALRISQSTFNNPRPGQLGRLLPNQNLPLDITLQSPTGAGPFPPIRNSSPYSVIPQPGMMG
NQGMIGNQGNLGNSSTGMIGNSASRPTMPSGEWAPQSSAVRVTCAATTSAMNRPVQGGMI
RNPAASIPMRPSSQPGQRQTLQSQVMNIGPSELEMNMGGPQYSQQQAPPNQTAPWPESIL
PIDQASFASQNRQPFGSSPDDLLCPHPAAESPSDEGALLDQLYLALRNFDGLEEIDRALG
IPELVSQSQAVDPEQFSSQDSNIMLEQKAPVFPQQYASQAQMAQGSYSPMQDPNFHTMGQ
RPSYATLRMQPRPGLRPTGLVQNQPNQLRLQLQHRLQAQQNRQPLMNQISNVSNVNLTLR
PGVPTQAPINAQMLAQRQREILNQHLRQRQMHQQQQVQQRTLMMRGQGLNMTPSMVAPSG
MPATMSNPRIPQANAQQFPFPPNYGISQQPDPGFTGATTPQSPLMSPRMAHTQSPMMQQS
QANPAYQAPSDINGWAQGNMGGNSMFSQQSPPHFGQQANTSMYSNNMNINVSMATNTGGM
SSMNQMTGQISMTSVTSVPTSGLSSMGPEQVNDPALRGGNLFPNQLPGMDMIKQEGDTTR
KYC

Figure S1: FASTA sequences of SRF (A), NCOA2 (B) downloaded from https://www.uniprot.org/ and the respective three-dimensional structures obtained starting from the FASTA sequences with the use https://swissmodel.expasy.org/interactive/S4rVBg/models/. (C) Amino acid sequence of the SRF-NCOA2 fusion protein obtained by joining the two parts of the sequences at the breaking point. The functional domains of each protein were highlighted: MADS-Box (red), DNA-binding (yellow), bHLH-domain (pink), FAS-domain (green), breackpoint (light blue).

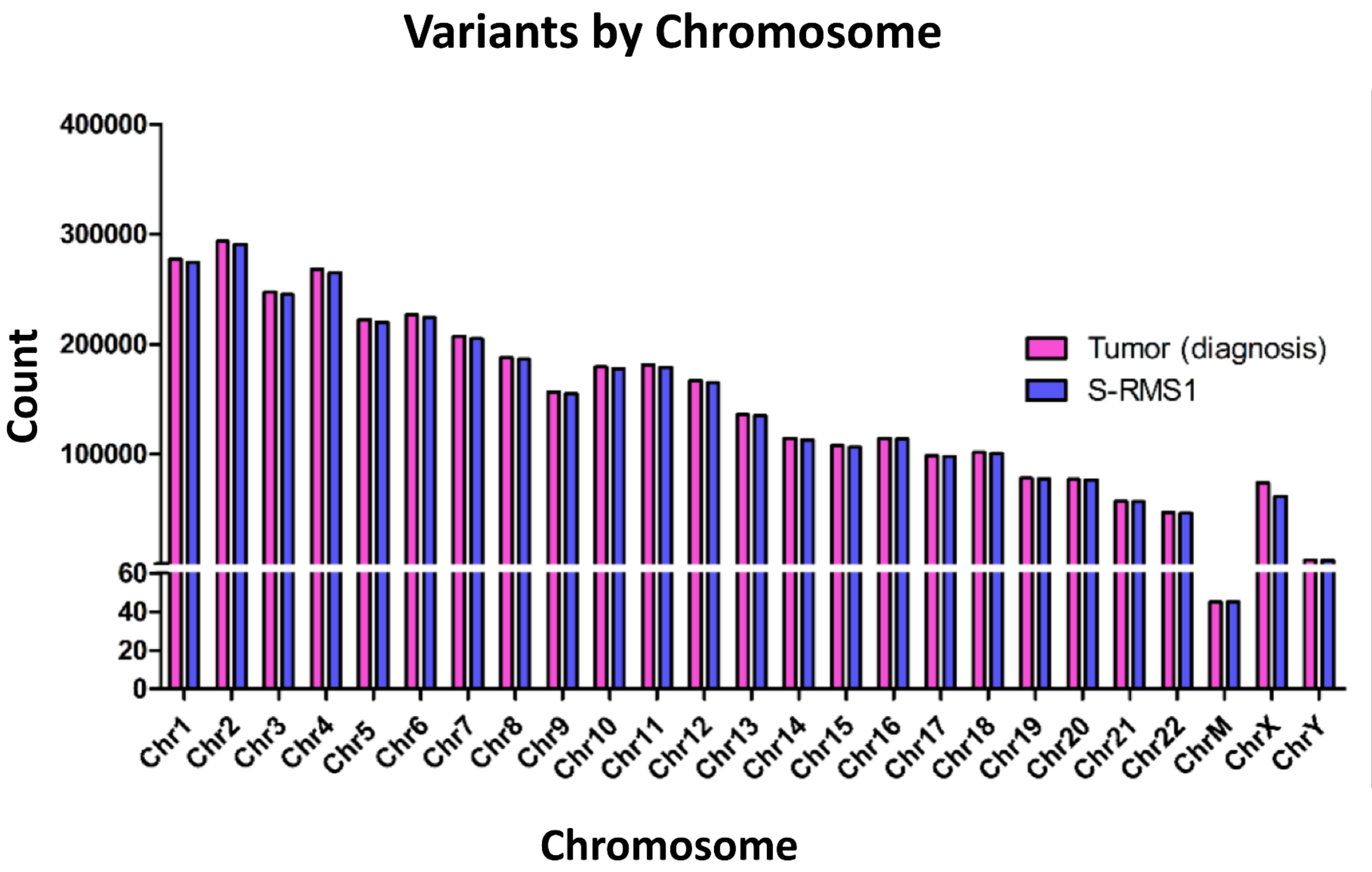

Figure S2: The histogram depicts the number of variant counts in each chromosomes of patient genome for tumor at diagnosis (pink bars) and S-RMS1 (blue bars) samples.

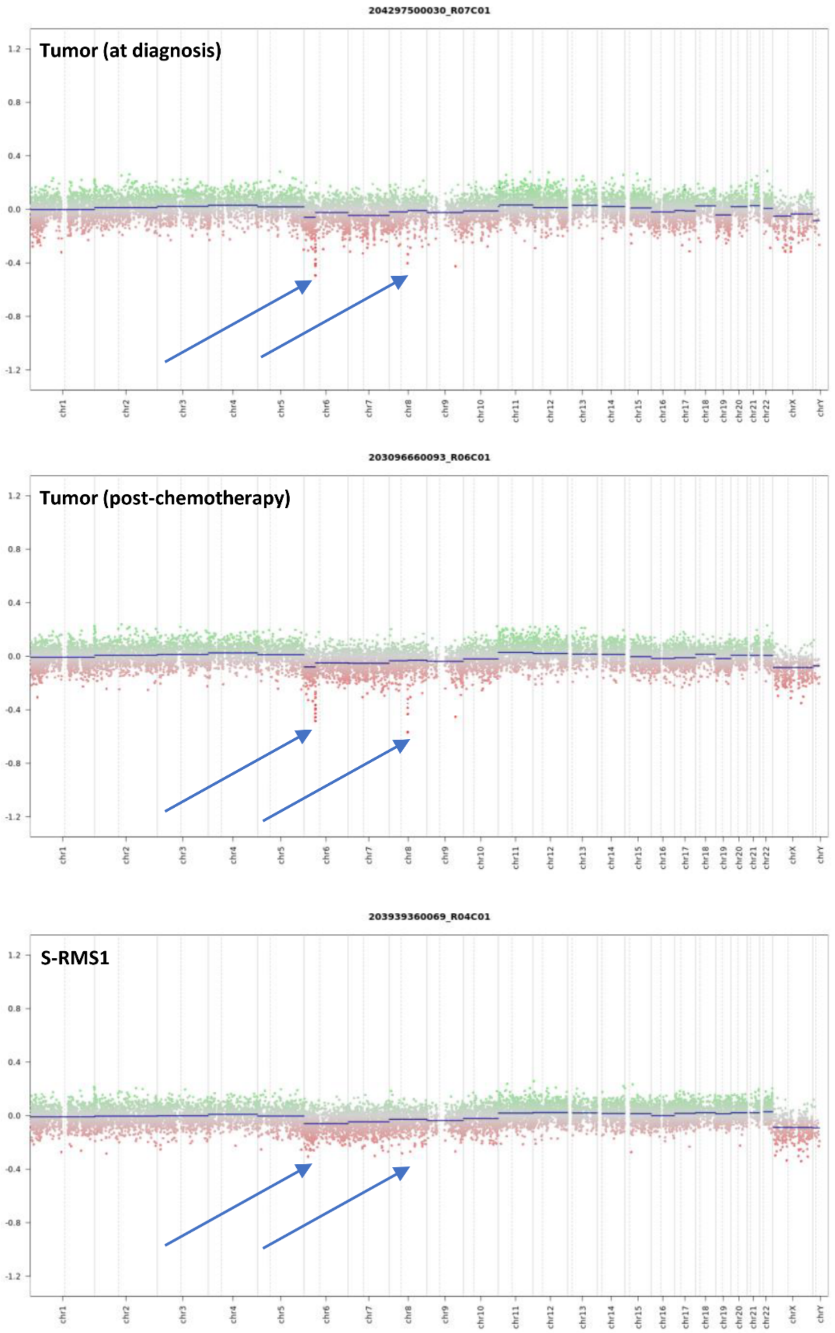

Figure S3: Copy number variation plot calculated from DNA methylation array data of the indicated tumor samples and cell line (S-RMS1). Depiction of structural rearrangements involving autosomes and X/Y chromosomes. Gains/amplifications represent positive (green), losses negative (red) deviations from the baseline. Blue arrows highlight chromosome 6p and 8q deletion corresponding to the breakpoints of SRF and NCOA2 respectively.

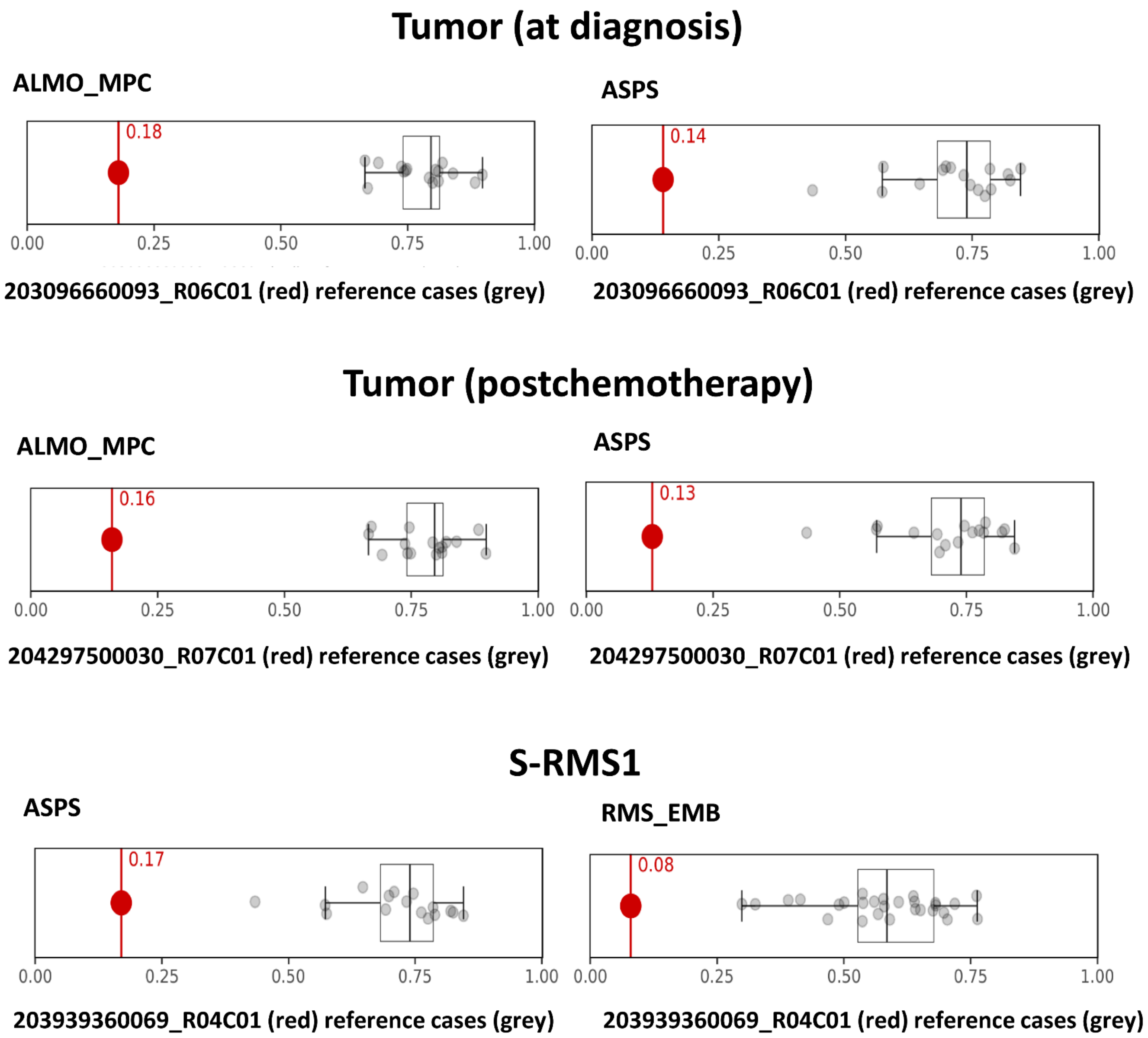

Figure S4: Box-and-whisker plots depicting the first and the second highest raw classification scores (red dot) of the tumor samples and cell line in the indicated methylation classes according to Sarcoma Classifier v12.2 (https://www.molecularneuropathology.org/mnp/classifier/9). Grey dots represent the reference cases in the methylation class. ALMO_MPC angioleiomyoma / myopericytoma; ASPS alveolar soft part sarcoma; RMS_EMB rhabdomyosarcoma, embryonal
